# Sharing Reliable COVID-19 Information and Countering Misinformation: In-Depth Interviews With Information Advocates

**DOI:** 10.2196/47677

**Published:** 2023-10-20

**Authors:** Alexis M Koskan, Shalini Sivanandam, Kristy Roschke, Jonathan Irby, Deborah L Helitzer, Bradley Doebbeling

**Affiliations:** 1 College of Health Solutions Arizona State University Phoenix, AZ United States; 2 Walter Cronkite School of Journalism and Mass Communication Arizona State University Phoenix, AZ United States

**Keywords:** COVID-19, coronavirus, pandemic, infodemic, misinformation, social media, qualitative research, public health, health communication

## Abstract

**Background:**

The rampant spread of misinformation about COVID-19 has been linked to a lower uptake of preventive behaviors such as vaccination. Some individuals, however, have been able to resist believing in COVID-19 misinformation. Further, some have acted as information advocates, spreading accurate information and combating misinformation about the pandemic.

**Objective:**

This work explores highly knowledgeable information advocates’ perspectives, behaviors, and information-related practices.

**Methods:**

To identify participants for this study, we used outcomes of survey research of a national sample of 1498 adults to find individuals who scored a perfect or near-perfect score on COVID-19 knowledge questions and who also self-reported actively sharing or responding to news information within the past week. Among this subsample, we selected a diverse sample of 25 individuals to participate in a 1-time, phone-based, semistructured interview. Interviews were recorded and transcribed, and the team conducted an inductive thematic analysis.

**Results:**

Participants reported trusting in science, data-driven sources, public health, medical experts, and organizations. They had mixed levels of trust in various social media sites to find reliable health information, noting distrust in particular sites such as Facebook (Meta Platforms) and more trust in specific accounts on Twitter (X Corp) and Reddit (Advance Publications). They reported relying on multiple sources of information to find facts instead of depending on their intuition and emotions to inform their perspectives about COVID-19. Participants determined the credibility of information by cross-referencing it, identifying information sources and their potential biases, clarifying information they were unclear about with health care providers, and using fact-checking sites to verify information. Most participants reported ignoring misinformation. Others, however, responded to misinformation by flagging, reporting, and responding to it on social media sites. Some described feeling more comfortable responding to misinformation in person than online. Participants’ responses to misinformation posted on the internet depended on various factors, including their relationship to the individual posting the misinformation, their level of outrage in response to it, and how dangerous they perceived it could be if others acted on such information.

**Conclusions:**

This research illustrates how well-informed US adults assess the credibility of COVID-19 information, how they share it, and how they respond to misinformation. It illustrates web-based and offline information practices and describes how the role of interpersonal relationships contributes to their preferences for acting on such information. Implications of our findings could help inform future training in health information literacy, interpersonal information advocacy, and organizational information advocacy. It is critical to continue working to share reliable health information and debunk misinformation, particularly since this information informs health behaviors.

## Introduction

In the United States, the COVID-19 pandemic and the resulting “infodemic” that ensued highlighted the role of the rampant spread of misinformation and its impact on health-related decisions [1]. Survey research conducted in November 2021 by Kaiser Family Foundation found that nearly 80% of respondents believed a common myth about COVID-19 or COVID-19 vaccines (eg, the vaccine causes infertility and the government exaggerated COVID-19 deaths) or were unsure about the accuracy of such myths [2]. Widespread misinformation, in turn, impacted COVID-19 prevention behaviors. For example, individuals exposed to misinformation about COVID-19 vaccines were more likely to reject the evidence-based recommendation of receiving the vaccine [3].

In understanding the spread of misinformation, it is critical to explore which information sources people trust and distrust and which sources they use to find reliable health information. Before the COVID-19 pandemic, people generally trusted medical and scientific sources for their health information. However, Pew Research recently published a report illustrating the waning trust in various authorities, including scientists and medical professionals, with more adults reporting lower levels of trust since the beginning of the pandemic [4]. At a closer look, the levels of trust in science differed based on political party affiliation, with Republicans reporting reduced trust while Democrats reported the same level of trust in scientific authorities [4]. Further, believing COVID-19 misinformation was influenced by which national news media individuals regularly watch or read, with more individuals believing misinformation when they watched or read conservative network news (eg, Fox News and Newsmax) [2].

It is critical to explore how some individuals found correct information about the pandemic at a time when misinformation was widespread. Such strategies can be used for future training in information and media literacy. Additionally, assessing how these individuals with accurate information acted as information advocates, sharing reliable COVID-19 information and combating or debunking misinformation, is crucial. Information advocates are expected to have uncovered effective practices to avoid COVID-19 misinformation and share reliable COVID-19 information. Therefore, the purpose of this study was to explore well-informed (about COVID-19) information advocates’ health information sources and behaviors, including their practices of disseminating accurate, trustworthy COVID-19 information and combating misinformation about the pandemic.

## Methods

### Overview

In order to recruit our sample, we selected individuals who completed our previous internet-based survey, which was hosted on QuestionPro (Survey Analytics LLC) and disseminated via an internet-based research panel in September 2022, about COVID-19 and scored a perfect or near-perfect score on COVID-19 knowledge questions and who actively shared or responded to news information within the past week. The 18 knowledge-based survey items about COVID-19 included 8 questions taken from the Kaiser Family Foundation’s survey, which assessed adults’ beliefs in common COVID-19 myths [2]. Additionally, 1 team member (an infectious disease epidemiologist) created 20 questions about the safety and effectiveness of COVID-19 prevention and control strategies. A team consisting of an epidemiologist, an emergency medicine physician, a physician specializing in infectious diseases, and a public health professor reviewed these 20 survey items, and to reduce the burden on the participants, recommended including 10 of these questions. Multimedia Appendix 1 lists the final 18 COVID-19 knowledge questions. Further, to identify individuals who actively share information, we included 2 questions that asked participants about how they shared or responded to news information within the past week.

A total of 1498 individuals completed the survey, of whom 603 (40.3%) scored 18/18 correctly, and 261 (17.4%) correctly answered 17/18 COVID-19 knowledge questions. Of these 864 individuals, 765 (88.5%) reported having acted on (eg, sharing, liking, discussing, and debunking) news information within the past week, of whom 486 (63.5%) provided contact information for a follow-up interview. We selected a diverse sample of these individuals to complete interviews for this study and conducted all interviews in October 2022.

We created and pilot-tested a semistructured interview guide with 2 COVID-19 experts from the aforementioned team of scientific and medical experts (see Multimedia Appendix 2 for the interview guide). We messaged participants within 1 month of their survey completion to invite them to participate in a 1-time interview and schedule it. The first author texted all potential participants who had scheduled an interview and reminded them of the upcoming phone interview using Google Voice (Google LLC). On the day of the interview, she called the participants, described the study, obtained their informed consent, and conducted the interview. She emailed all interview participants a US $30 Amazon e-gift card as compensation for their time and shared experiences. All interviews were recorded, and a professional transcription service transcribed the audio files.

Using an inductive approach, 1 study team member read all transcripts and served as the primary codebook creator and editor, expanding and merging codes as needed. To enhance the rigor of the qualitative analysis, she trained another study team member to coconduct the thematic analysis [5]. They met weekly to discuss coding guide definitions, update the coding guide as needed, and reach a consensus on how they coded interview data. They first coded 1 transcript together to test the coding guide and ensure coding similarities. Then, they coded 3 transcripts separately before meeting and resolving coding discrepancies and making further clarifications to the coding guide (expanding code definitions, merging codes, etc). They coded 2 additional transcripts separately before reaching a consensus on coding and splitting the remaining transcripts to hand-code and enter into ATLAS.ti software (ATLAS.ti Scientific Software Development GmbH). They aggregated the ATLAS.ti output, synthesized their findings, and summarized the results by code. Finally, they created a comprehensive summary of the findings, identifying quotes that best illustrated emerging interview themes and subthemes.

### Ethics Approval

The Arizona State University Institutional Review Board approved this research study (STUDY00015977).

## Results

### Overview

The in-depth interview participants represented a diverse sample of 25 US adults of various ages, races, communities (eg, rural, suburban, and urban), and political affiliations. It is important to note that all participants reported vaccinating against COVID-19, although this was not part of the inclusion criteria for the study. See Table 1 for participants’ demographic information.

In their interviews, information advocates described the sources of information they trusted for COVID-19 information, the sources they did not trust, their processes for determining information credibility, responses to misinformation, and how they shared reliable COVID-19 information. See Table 2 for themes and subthemes that emerged from the qualitative data and representative quotes that illustrate these findings.

**Table 1:** Demographic information of interview participants (N=25).

Interview participant demographics		Values
Age (years), mean (SD)		46.1 (13.5)
**Sex, n (%)**		
	Female	10 (40)
	Male	15 (60)
**Ethnicity, n (%)**		
	Hispanic	4 (16)
	Non-Hispanic	21 (84)
**Race or ethnicity, n (%)**		
	White	17 (68)
	Hispanic	3 (12)
	Black	2 (8)
	Asian	3 (12)
**Education, n (%)**		
	Some high school	1 (4)
	Some college	3 (12)
	Trade or tech school	2 (8)
	College graduate	10 (40)
	Some postgraduate work	4 (16)
	Postgraduate degree	5 (20)
**Employment, n (%)**		
	Full-time	12 (48)
	Part-time	3 (12)
	Retired	3 (12)
	Not employed	6 (24)
**Income (US $), n (%)**		
	$0-$25,999	4 (16)
	$26,000-$51,999	6 (24)
	$52,000-$75,000	2 (8)
	$75,000-$100,000	6 (24)
	$100,000-$200,000	6 (24)
	≥$200,000	1(4)
**Political affiliation, n (%)**		
	Democrat	14(56)
	Republican	3 (12)
	Moderate	1 (4)
	Independent	7 (28)
**Community, n (%)**		
	Rural	5 (20)
	Town	4 (16)
	Suburb	8 (32)
	Large city	8 (32)
**Insurance status, n (%)**		
	Insured	24 (96)
**PCP^a^ status, n (%)**		
	Has a PCP	24 (96)
**COVID-19 vaccines received, n (%)**		
	Initial doses and booster dose	21 (84)
	1+ doses of a COVID-19 vaccine (initial doses only)	4 (16)
**Previous COVID-19 diagnosis, n (%)**		
	Yes	8 (32)
	No	17 (68)

^a^PCP: primary care provider.

**Table 2:** Qualitative responses illustrating the study findings.

		Example quote	
**Trusted information source**			
	Neutral information sources	Yeah, not Fox News or MSNBC. Just something neutral. (Participant 23)	
	News media	Well, even though I don’t own a TV, I have a laptop and what I do is go to YouTube and I’ll watch certain news programs, like PBS NewsHour. I’ll watch from the three major networks CBS, NBC, and ABC, their nightly news broadcasts on YouTube. Also, on my phone, I look at the Apple News app, and I have programmed in there what periodicals I want to look at. So, it might be the New York Times I have on there, the Washington Post, maybe USA Today’s on there. (Participant 21)	
	State and local news (as opposed to national news)	I actually found the New York State Governor’s press conferences very informative. I also used the news for my county. I live in upstate New York, [and I look at] their public health Facebook accounts just to keep track of the local trends and statistics. They were very good at updating those regularly. I’m so far away from New York City that we had a totally different wave and a totally different behavior of COVID up here. So, if you were listening just to New York City and the trauma they had down there, you would have a totally different view of what it was like up here.... There were a lot of restrictions put on upstate New York when there didn’t need to be. And many things happened in New York City that didn’t apply to upstate New York. (Participant 18)	
	Monitored sources	Whenever it first started, I originally started reading about it on Reddit. And as it started spreading to the U.S. Then I would follow it on a Reddit Megathread that always had updated information. (Participant 7)	
	Health care provider	Before the shots came out, I talked with doctors who were struggling to find how to fix it. I watched the news sometimes, but mostly, I listened to the doctors who were helping my dad. That was really about it. Just listening to the doctors and the pharmacist. (Participant 14)	
**Untrusted sources**			
	Pseudoscience	Sometimes you’ll see a really sketchy URL in the search results that seems hokey or not really science-based, more like a holistic medicine or like the woo-woo stuff that you can pretty much tell from the link and the URL that it’s pseudoscience. (Participant 5)	
	Social media	All these platforms that provide information, such as social media, do a very poor job of giving you proper information. And they’re so fantastic at throwing you bad information without any censorship or warning. We seem to have a culture that promotes misinformation based on how viral it [the information] could be or how emotionally appealing it can be. When it comes to information, if you’re going into emotions, first, you have to be drawn to something that correlates more to your original thought process than the actual truth of the situation. (Participant 20)	
	News media	Take everything with a grain of salt when you’re watching the news. Don’t take in everything at face value, I guess. (Participant 14)	
**Deciding if a source is trustworthy**			
	Data-driven sources	Yeah. I mean, I feel like certain sources are more data-based. They’re less speculative. They run more on actual research and science and data. I know that the Mayo Clinic is like that, and they cite research and specific studies. Things that make them more vetted and reliable as a source of information. The CDC, I know, is data-based and research-based, so I just feel like they’re more credible. (Participant 5)	
	Trust in scientific professionals	These people spend their entire lives - doctors, scientists, and researchers - these people spend their whole lives trying to better humanity, protect us from diseases, and improve our wellness. You need to extend some trust to these people. (Participant 2)	
	Assess information and sources for bias and a hidden agenda	Lots of times misinformation will be tied to some third-party websites or certain websites funded by certain think tanks or certain groups that are very political-leaning one way. So, I can usually get an indication of where this information comes from and what kind of political agenda it has. (Participant 19) Yeah, I would say get the actual source of the information. Most information has quotes compiled from people which are usually compiled from other sources. So, I would say first, find the source of the information that you’re receiving. And I would say secondly, find out who is funding the people getting the information and what their intentions may be. So, I think you know in the short term, follow the money. Follow the information and follow the money. Get a good idea as to what initiative has brought you that information. (Participant 20)	
	Check the information with multiple sources	Check for multiple sources to get a similar result, a similar answer to what you’re looking for. Check multiple sources because if it’s out there in one source, it doesn’t mean it’s [the information] true. So, I would just tell them to check. Like if you’re unsure, check, and then if you find it, check again because that first one could be an offshoot, and then just maybe three or four [sources]. And think after three or four [sources], you probably have a good idea that it’s well-reported and probably legitimate. (Participant 22)	
	Fact-checking software	I would say if you find something or if you hear something, utilize one of the fact-checking sites. This is what I was doing early on, too. You know, the fact-checking software, like Snopes - just to kind of see if something is true or false. (Participant 8)	
**Responses to misinformation**			
	Ignore misinformation	If I see the headline and it’s something that I think is absolutely incorrect, I already know the facts, so I don’t think I bother with it. (Participant 12) I know I’ve seen videos on YouTube in my feed that are undoubtedly misinformation or lies, and I try to ignore them. I don’t have any process for reporting or logging them or anything like that. I try to just filter it out and go about my business. (Participant 5)	
	Correct misinformation	Well, I hear the information, like okay. I mean, I’m trying to see it from their point of view because I’m not sure I’m just being speculative from my point of view. Like, are they just hesitant? Are they afraid about the outcome of it? And just try to project their opinion and try to spin it in their own sort of way that fits their narrative. But I don’t really do anything. I just think, “Okay, that’s your opinion.” I don’t leave comments. I don’t “like” it. I don’t “dislike” it [referring to the action of responding to information with emojis]. I just move on. (Participant 24)	
	Action depends on the relationship to the person posting misinformation	My head goes to social media, where I see a lot of misinformation. It depends on the person as to whether I confront them, like if I was close enough to them. But I would try to back up what I am saying if I were to confront somebody about it. But otherwise, I would just ignore the articles and stuff people posted. (Participant 25) I don’t know whether there are a lot of people like me. I just kind of like back away. I don’t want to deal with this. Or, I don’t want to potentially ruin a friendship or alienate a family member. (Participant 11) So, if it’s a friend or a family member, then, of course, I’ll tell them [that their information is inaccurate]. But let me rephrase that. If it’s a friend or a family member on my dad’s side, I’ll make sure to correct them about it. If it’s a family member on my mom’s side, they are absolute loons. My dad and I tried to talk to them about it, but they just absolutely would not hear it. They were these people who thought COVID was made up to influence the election. They also thought 5G networks caused it, even though 5G towers don’t even exist where we live. There was just so much nonsense, of the fake information of where I currently lived when the pandemic started, that it was just insane. There was just no talking to them. (Participant 15)	
	Flagging or reporting misinformation	I believe that there’s a lot of misinformation, but what I’ve done is I’ve sent in like a flag or something to that effect or tried to come up - not come up, but present a fact that - an actual fact versus whatever the misinformation is. (Participant 3)	
	Blocking misinformation	If it’s online, it depends on how badly it upsets me. Sometimes if I’m on Twitter, I will tweet it and say, “Yeah, no, that’s not right. Here’s better information.” If it’s just something that I thought I should follow, then I will immediately unfollow and block [the account that posted misinformation] and move on. I find there’s a lot of shouting into the void on Twitter, and I’m not as participatory on Facebook. (Participant 18)	
	Regret over confronting misinformation	I mean, I had a few experiences where people who were sharing misinformation were really defensive about their bad information. There was one guy who, I forget when this was, maybe halfway through, a little bit before halfway through. He posted on Facebook, and he said, “This is nothing but the flu.” And I put a comment saying, “That’s reckless. You could be hurting people by putting this kind of stuff out there. You should be more careful about what you’re saying.” And then, this person started sending me private messages, demanding an apology for insulting him. Yeah, I kind of think in a lot of cases, it’s not worth messing with crazy. (Participant 11)	
**Sharing reliable health information**			
	Direct forms of interpersonal communication	I basically would tell them, like my mom. And some calls and texts to my friends. If it was a person that I don’t know, I would text them. The people I do know, I would tell them in person. And I would also text and email them. But primarily, it was a phone call or a text. (Participant 13) I can tell them what I know. It’s about all I like to do with that, and I guess I judge by the tone of the conversation if they want to hear it or not. (Participant 6) It’s usually just talking with neighbors and friends, just talking about it. (Participant 17)	
	Social media posts	I usually post it on my Facebook page or my Twitter page. And if it’s like really good, I’ll pin it to the top. So, when the county started detecting COVID in wastewater reports, I thought that was really interesting. They could predict what they would see in a couple of weeks in the hospitals [based on what they detected in wastewater]. So, I thought that was really interesting. (Participant 22)	
	Resharing information	Online, I would retweet tweets or post on Facebook things that ultimately found reliable. And things like, well, these are the statistics; this is the delta strain; this is what we’re trying to deal with right now. Things would have to meet certain criteria for me to retweet it or post it on Facebook. But I feel like some of the people that I talked to aren’t as savvy when it comes to scientific literacy. And I don’t want to accidentally push them the wrong way. So, I have to have a level of care when I try to promote it [information]. (Participant 17)	

### Trusted Information Sources

Participants trusted numerous science- and data-driven sources for their COVID-19 information, including scientific professionals such as physicians and pharmacists. Most often, participants cited trusting specific scientific organizations such as the US Centers for Disease Control and Prevention (CDC), the World Health Organization (WHO), and Johns Hopkins for their up-to-date information on the pandemic. Some used these sources to cross-verify information from other sources.

An equal number of participants reported relying on national and local news for their up-to-date information about COVID-19. Among those who reported trusting national news, they received this news information from mainstream television news, newspapers, or articles on news aggregator apps and believed that these news sources also allowed them to understand the global and national scope of the pandemic. Others described trusting state and local governments and health departments to provide the latest updates about COVID-19 and COVID-19 vaccines. Some described information shared on national news as misleading, part of a political agenda, or not representative of what was happening in their local communities. Especially as mandates on social distancing and masks varied by county, some reported relying more heavily on local news.

Participants who trusted social media platforms for their information stated that they followed credible sources or accounts on these sites. For example, many participants followed medical or health professionals’ accounts on Twitter. They believed they could differentiate accurate from inaccurate information and also trusted these specific experts’ information. Another commonly used social media site was Reddit. Participants described using the Reddit news feed for receiving COVID-19 headline news and trusted specific pages on Reddit (subreddits) whose users engaged in monitoring practices for information posted on the page.

### Untrusted Sources

Participants described a lack of trust in information shared on some social media sites, particularly Facebook. They saw this site as one where people more often shared opinions and emotionally-charged information instead of facts or fact-based articles. Further, participants noted how misinformation spreads quickly on social media sites since users, many of whom are not health or science experts, can post or share information. These nonexperts propagated news from unverified sources, particularly misinformation that appealed to people’s emotional and fear-based states.

### Deciding Whether a Source Is Trustworthy

Participants described their processes for deciding whether certain information is credible and trustworthy. They discussed the importance of examining the sources who posted information about COVID-19. Participants also noted their trust in science and individuals trained in scientific disciplines, particularly since they had dedicated their profession and lives to health and science.

When asked what made a source reliable or trustworthy, participants described using data-driven sources that provided statistical information on COVID-19 incidence, prevalence, infection rate, hospitalizations, and mortality. When they questioned information about the pandemic, participants described examining the source for their potential bias or for a hidden agenda. Some participants described how, upon hearing certain claims, they would examine the source to identify a political agenda or a potential financial gain for posting such information.

Most participants described the need to verify information when they were uncertain of the information’s trustworthiness. Often, they cross-referenced the information to either a trustworthy source or information provided by multiple sources. They described critically appraising information that did not align with what other sources were saying. Other times, they described verifying the information using fact-checking software. Participants also described verifying health information with their health care providers, given their expertise in health and medicine.

Some participants assessed whether the information they were exposed to on social media sounded plausible, using common sense to parse fact from fiction. They often provided specific examples of COVID-19 myths, such as chips implanted into individuals who receive COVID-19 vaccines (and tracked via the 5G network) and drinking bleach to help treat COVID-19. These participants recommended critical thinking as a means to reject health misinformation.

### Responses to Misinformation

The most commonly reported response to seeing or hearing misinformation was to ignore it. Participants who reported seeing misinformation on social media or web-based stories would scroll past the information, with some elaborating on how misinformation was a distraction and not worth exploring. Others explained how, out of curiosity, they were willing to listen to or read more of the misinformation without correcting the source. These individuals believed that correcting the misinformation was a poor use of their time, particularly if they sensed that the other party was adamant about their beliefs. Others described how the context of the relationship to the person posting or sharing misinformation would influence whether or not they responded. Some preferred ignoring information rather than confronting the individuals propagating misinformation to avoid online controversy and potentially ruin relationships in their social network.

Occasionally participants reported flagging and reporting web-based misinformation. Some participants blocked misinformation spread via social media and felt it was not helpful to address it, particularly on social media sites such as Facebook and Twitter. Others would alert the other person sharing misinformation that their information was inaccurate, posting the correct information as an online response. However, their willingness to correct misinformation depended on the type and closeness of the relationship to the person sharing misinformation. For example, some individuals felt more comfortable correcting misinformation with people with whom they shared a closer relationship or who shared similar beliefs. Others, regardless of the response they received or the context of their relationship, reported having confronted misinformation. They believed that the misinformation, especially if viewed by others, could negatively impact others’ health.

### Sharing Reliable Health Information

More participants were comfortable sharing reliable health information through in-person discussions or other direct forms of communication such as texting. Some noted elements of the in-person communication (eg, nonverbal communication) that influenced the amount of information they would share, whether they provided any clarification, and the extent to which they would discuss the pandemic.

Others created social media posts to share reliable and interesting COVID-19 information. Few participated in online groups, individuals who self-selected to be in a forum due to similar interests (eg, gaming), health conditions (eg, immunocompromised), or geographic community. Often, participants described vetting information to make sure it was correct and coming from a reliable source before sharing or resharing posts on social media or web-based news articles.

## Discussion

### Principal Findings

This research moved beyond past research that described the content of misinformation and characteristics of people who believe in misinformation [6,7] to examine the information-related practices among well-informed individuals who also act as information advocates. Findings describe not only the information sources that information advocates trust and distrust to find reliable health information but also the various ways they reacted to misinformation. Results illustrate how well-informed information advocates shared reliable information among members of their social network, both through direct interpersonal communication and on the internet, in more public forums. Results also describe the role of interpersonal relationships and the closeness of social ties when considering whether to respond to misinformation.

Information advocates discussed their trust in science, medicine, physicians, and public health organizations. They neither rejected science nor engaged in conspiratorial thinking, which are the traits of individuals who believe in health misinformation [8]. Instead, similar to past research, study participants preferred receiving COVID-19 information from individuals and organizations who had or represented relevant scientific training or background and whom they believed lacked a hidden agenda for sharing information about the pandemic [9]. It is critical to capitalize on these trusted relationships by offering training for health care providers to, at the interpersonal level, address patients’ misinformation. Further, at a community level, such experts can help organizations (including community-based organizations) develop and deploy health messaging [10].

Participants expressed mixed reviews about finding trustworthy information on social media. A few participants described trusting information on specific Twitter accounts and Reddit pages. These pages may have administrators, moderators, or followers who audit and regulate content and act against misinformation or individuals posting it. On the other hand, other information advocates noted their lack of trust in social media sites, particularly Facebook, a site where they believed that opinions and anecdotes, not facts, spread rapidly. Interestingly, a study conducted by Yang et al [11] found surges of low-credibility content posted on both Facebook and Twitter, with Facebook having a greater volume of low-credibility information in January 2020 and on Twitter between April and October 31, 2020, with Twitter posting more misinformation, overall. To combat misinformation, social media platforms heavily invested in content moderation and flagging systems; however, as the tech industry continues to face financial concerns and budget cuts, social media platforms are deprioritizing the fight against misinformation [12]. Passing legislation that requires social media platforms to remove misinformation is 1 systems-level approach that could be a critical step in addressing health-related misinformation on the internet, particularly at a time when their efforts are waning [12]. The US Department of Health and Human Services continues urging researchers to engage in multidisciplinary, multilevel research to identify ways to detect and combat the impact of misinformation, particularly among populations who experience health disparities (NIH RFA-MD-22-008).

Information advocates used a comprehensive range of sources to get the complete picture of the current COVID-19 pandemic state of affairs. They broadened how they searched for information, taking local, national, and global perspectives into consideration to understand the scope of the pandemic. They looked for data-driven sources and cross-referenced information with multiple sources. They questioned the motives of information sources, specifically looking for potential hidden agendas and ulterior motives, a practice recommended for identifying misinformation [13]. Information advocates described gauging the plausibility of the information and using common sense to assess the accuracy of information they heard about COVID-19. They reported their lack of reliance on their own intuitions and emotions to assess whether they believed COVID-19 information seemed correct, practices associated with believing false information and spreading misinformation [14]. Some also described using fact-checking sites to assess information accuracy, which can help individuals differentiate between accurate and inaccurate information. Using such sites and identifying web-based misinformation may lead to their debunking (correction of misinformation) of that information, a behavior signifying their interest in acting as information advocates as opposed to information consumers, alone [15]. The National Association for Media Literacy Education recommends using all of these strategies to encourage critical thinking about information posted on the internet [16]. Instilling critical thinking and media literacy skills may enhance individuals’ ability to detect misinformation. However, little agreement exists about the best methods (eg, training courses and gamification of information) to enhance media literacy in a “generationally inclusive manner” [17].

Some information advocates described specific actions (eg, flagging and reporting misinformation) to alert websites to user accounts that were actively spreading inaccurate information. Other times, participants blocked users who spread misinformation. Previously, science communication experts assumed that misinformation is spread because people lack access to factual information or the tools to discern fact from fiction, known as the knowledge deficit model [18]. However, behavioral researchers argue that misinformation is accepted as true and then spread, not as a result of mere ignorance but due to psychological factors such as conspiratorial mentality, individual fears, identity expression, and motivated reasoning (driven by personal or moral values over objective evidence) [19]. They therefore recommend that information advocates respond to misinformation in the following three ways: (1) directly identify misinformation and counter it with fact-based information, (2) identify and address the fallacies in the misinformation sharer’s argument, and (3) question the misinformation and source’s credibility [19]. Further, to avoid unintentionally sharing misinformation, researchers recommend “nudging” individuals to assess the accuracy of information before they share it on the internet, a practice that, in 1 experiment, enhanced social media users’ intentions to engage in critical thinking prior to sharing information within their social networks [20].

Participants were concerned that challenging misinformation could negatively impact their interpersonal relationships. In considering the spiral of silence communication theory, most people prefer to remain silent when their opinion deviates from the dominating view rather than refuting or challenging misinformation [21]. Ignoring misinformation and remaining nonresponsive to it may help them maintain their online reputation or help them avoid violating the norm of online politeness [22]. Some felt more comfortable correcting misinformation with individuals with whom they perceived their social connection to be secure. This is consistent with previous work that illustrated how individuals were more likely to correct misinformation or share debunking information if the original source of that information was a close tie and someone who shares similar traits (in-group members) [23]. Past research suggests that misinformation correction may be more acceptable (and perceived as a less aggressive form of communication) if the relationship with the person being corrected is strong [24]. Correcting misinformation with a weaker tie may further compromise and weaken their social connection [24]. Future research can identify strategies that allow for correcting misinformation in a way that does not weaken interpersonal relationships. Further, future research can identify ways to increase social norms about correcting misinformation on the internet [25]. Additionally, trusted organizations and experts can engage more actively in correcting misinformation [25], especially because doing so does not negatively influence the organizations’ credibility or relationships [26]. The US surgeon general further recommends professional associations to provide information or training to their members to serve as experts in sharing and correcting information, including on web-based forums [10]. Such misinformation correction, particularly when made by expert sources, can lead to observational correction, when other social media users change their own attitudes or understanding about a topic after clarification or correction has been publicly made on the internet [26]. Although past research has warned about a backfire effect (originally used in political science), when individuals strengthen their original belief in misinformation after hearing a counterargument, the prevalence of this effect can be exaggerated and is, in fact, most common among individuals who hold more extreme beliefs about particular topics [27].

Some information advocates based their decision to correct web-based misinformation on how consequential they perceived believing in or acting upon the misinformation could be, acting as “communal guardians” of information. They preferred violating the norm of politeness (by not correcting the misinformation) if the outcome meant protecting other information consumers [22]. For example, 1 participant described deciding to confront misinformation based on her emotional response (outrage) to the misinformation. Although past research describes how web-based misinformation often evokes a strong emotional response, which encourages readers to share this information [14], our study finds that web-based misinformation prompted a similar reaction, leading 1 participant to debunk false information.

### Limitations

This study is not without its limitations. Our study only examined the perspectives of individuals with a perfect or near-perfect score on COVID-19 knowledge questions and who acted on news information within the past week. Because of this, we cannot speculate on the media literacy behaviors of those with lower COVID-related knowledge scores. Additional limitations are related to our sample, including the study’s small sample size. However, the study reached information saturation, a standard method for determining sample sizes in qualitative research. A strength of our sample was its diversity, with participants representing various ages, educational levels, household incomes, and other demographic factors. However, the majority of our nonprobability sample identified as White (n=17, 68%), highly educated (n=19, 76% had at least a college education), and Democrats (n=14, 56%) and therefore did not necessarily represent the diverse US population. This is a common problem among research panel participants, particularly MTurk [28]. There was little diversity in vaccination status; all participants received at least the original COVID-19 doses. However, this may be expected among people who reported trusting science and having high levels of COVID-19 knowledge. There was also less diversity in political ideology represented. This may be because Democrats demonstrated greater vaccine acceptance [29]. On the other hand, in individuals with conservative political leanings, the perceived threat because of COVID-19 was low and had lower vaccine acceptance [30]. Further, the original survey questions used to recruit our sample focused on sharing news information, not necessarily COVID-19 news. However, research illustrates that people share information and react to misinformation similarly regardless of its content [20]. Another limitation of qualitative interviews can include social desirability bias, when participants answer questions in a way that they believe is socially acceptable [31]. The interviewer used strategies to minimize such bias by hosting telephone interviews (instead of video-based interviews), describing the purpose of the study, discussing their anonymity, and assuring participants that there were no wrong responses.

### Conclusions

In this study, information advocates trusted science and scientific sources for their COVID-19 information. They preferred getting their information about the pandemic from renowned public health sources such as the CDC and WHO and also trusted social media accounts of health and scientific professionals. For other populations (who may not be as knowledgeable about COVID-19 or who do not act as information advocates), improving the credibility of government and health care institutions and their ability to share public health messages promptly and transparently is critical. Further, this research illustrated the importance of providing objective, nonpolitically biased health information. Future information and media literacy training can teach effective strategies for assessing the credibility of health information by prompting individuals to not only examine the sources but also to question the motives of those posting information. Other skills involved in information and media literacy training can emphasize similar skills used by our information advocates, such as cross-referencing information, using fact-checking sites to verify information, and talking to health care providers about any questions they have about health information.

Insight from this research illustrated how individuals who are well-informed about COVID-19 and serve as information advocates find, share, avoid, and confront misinformation about COVID-19. More individuals preferred sharing accurate information than countering misinformation, especially on the internet. Our findings describe how participants’ perceptions of the type and quality of their interpersonal relationships influenced their willingness to combat misinformation. They addressed web-based misinformation by flagging or reporting it. Offline, they preferred addressing misinformation in a more interpersonal and private manner, such as through in-person conversations or text messaging. Some information advocates feared losing relationships over correcting misinformation, while others, based on their perceptions of how dangerous it could be, perceived addressing such false information as an essential action to help protect members of their social networks.

Populations must have accurate health information about the pandemic and practice media literacy and critical thinking skills, particularly since this may affect their disease-prevention behaviors. Implications of our findings could inform future training in health information literacy, interpersonal information advocacy, and organizational web-based information advocacy. Imparting such skills through media and information literacy training may help others share credible, trustworthy information and avoid misinformation, leading to a more informed and healthier public.

## Appendix

Multimedia Appendix 1 COVID-19 knowledge questions used for selecting study participants.

Multimedia Appendix 2 Semistructured interview guide.

## Abbreviations

CDC: US Centers for Disease Control and Prevention
WHO: World Health Organization
