# Supplementary material for: Sharing Reliable COVID-19 Information and Countering Misinformation: In-Depth Interviews With Information Advocates
Source: JMIR Infodemiology. 2023 Oct 20;3:e47677. doi: 10.2196/47677 (PMC10625073; doi:10.2196/47677)
Supplement: Multimedia Appendix 1 [file infodemiology_v3i1e47677_app1.docx]

COVID-19 knowledge questions used for selecting study participants.

| 1) The COVID-19 vaccines (Pfizer, Moderna) are safe for most recipients. (True) |
| --- |
| 2) The COVID-19 vaccines (Pfizer, Moderna) are effective in preventing hospitalization. (True) |
| 3) The COVID-19 vaccines (Pfizer, Moderna) are effective in preventing death. (True) |
| 4) Regular use of masks in high-risk settings will lessen your risk of developing or spreading  COVID-19. (True) |
| 5) Avoiding close contact with others who have been exposed to or are sick with COVID is a  key strategy in preventing COVID-19. (True) |
| 6) Those who are fully vaccinated cannot transmit COVID-19. (False) |
| 7) The use of masks in schools has not been shown to reduce the risk of COVID-19 infection in  children. (False) |
| 8) COVID-19 can only be spread by those who are exhibiting symptoms. (False) |
| 9) Those who have had COVID-19 cannot contract the disease again. (False) |
| 10) Social distancing (staying 6 feet away from others) can help prevent the spread of COVID.  (True) |
| 11) The government is exaggerating the number of COVID-19 deaths. (False) |
| 12) Pregnant women should not get the COVID-19 vaccine. (False) |
| 13) The COVID-19 vaccines have been shown to cause infertility. (False) |
| 14) You can get COVID-19 from the vaccine. (False) |
| 15) The COVID-19 vaccines can change your DNA. (False) |
| 16) People of all ages can become infected with COVID-19. (True) |
| 17) People of all racial and ethnic groups can become infected with COVID-19. (True) |
| 18) Most people who are infected with the COVID-19 virus recover from it. (True) |
