# Supplementary material for: Sharing Reliable COVID-19 Information and Countering Misinformation: In-Depth Interviews With Information Advocates
Source: JMIR Infodemiology. 2023 Oct 20;3:e47677. doi: 10.2196/47677 (PMC10625073; doi:10.2196/47677)
Supplement: Multimedia Appendix 2 [file infodemiology_v3i1e47677_app2.docx]

Semistructured interview guide.

| Thank you for taking the time to take part in this interview. You have been identified as an individual who is very well-informed on COVID-19 information based on your responses to our previous survey research. These interviews will be used to get a better understanding of how people have found and made sense of reliable health information about COVID-19 prevention. We expect to use the findings from this research to develop approaches to spread the best practices we find here to improve public health and prevention. |
| --- |
| Since the beginning of the pandemic, what sources of information have you used to find reliable COVID-19 information? |
| How did you know that this reference was a reliable source to trust? |
| What has been your process of finding and interpreting information about COVID-19 vaccines? |
| How has your process for finding reliable information about COVID-19 vaccines or COVID-19 been different than finding other health information in the past? |
| What do you do when you find health misinformation? |
| How have you shared reliable health information with people in your social networks (in-person and/or online)? |
| During this time of rampant misinformation, what would you recommend to others – to find and analyze reliable health information? |
| Is there anything else you would like to share about your beliefs about finding information about COVID-19? |
| Thank you for taking the time to speak with me about your practices in identifying reliable COVID-19 vaccine information. I certainly appreciate your valuable input. This ends our interview. |
